# Protection and Virus Shedding of Falcons Vaccinated against Highly Pathogenic Avian Influenza A Virus (H5N1)

**DOI:** 10.3201/eid1311.070705

**Published:** 2007-11

**Authors:** Michael Lierz, Hafez M. Hafez, Robert Klopfleisch, Dörte Lüschow, Christine Prusas, Jens P. Teifke, Miriam Rudolf, Christian Grund, Donata Kalthoff, Thomas Mettenleiter, Martin Beer, Timm Harder

**Affiliations:** * Freie Universität, Berlin, Germany; † Federal Research Institute for Animal Health, Greifswald-Insel Riems, Germany

**Keywords:** Keywords: Birds of prey, falcon hybrids, highly pathogenic avian influenza, challenge, antibody titer, histopathology, H5N1, zoonosis, human risk, fowl plague, virus shedding, research

## Abstract

Because fatal infections with highly pathogenic avian influenza A (HPAI) virus subtype H5N1 have been reported in birds of prey, we sought to determine detailed information about the birds’ susceptibility and protection after vaccination. Ten falcons vaccinated with an inactivated influenza virus (H5N2) vaccine seroconverted. We then challenged 5 vaccinated and 5 nonvaccinated falcons with HPAI (H5N1). All vaccinated birds survived; all unvaccinated birds died within 5 days. For the nonvaccinated birds, histopathologic examination showed tissue degeneration and necrosis, immunohistochemical techniques showed influenza virus antigen in affected tissues, and these birds shed high levels of infectious virus from the oropharynx and cloaca. Vaccinated birds showed no influenza virus antigen in tissues and shed virus at lower titers from the oropharynx only. Vaccination could protect these valuable birds and, through reduced virus shedding, reduce risk for transmission to other avian species and humans.

## Abstract 2

Virus shedding by vaccinated birds was markedly reduced.

Highly pathogenic avian influenza A (HPAI) virus poses a major threat to poultry but is also of great concern for other avian species and humans. In particular, HPAI (H5N1) of Asian lineage is known for its potential to be transmitted to mammals, including humans. Susceptibility to this virus and the possible role as vectors or reservoirs vary greatly between different wild bird and poultry species (*1*,*2*). Gallinaceous poultry are considered to be highly susceptible, whereas waterfowl may show variable clinical signs depending on the strain of infecting virus. Birds of prey are at increased risk for infection with HPAI virus because they regularly feed on avian carcasses and diseased avian prey (*3*,*4*). Many species are migratory or cover an extensive territory and may spread the virus within or between countries. In falconry, birds of prey are also regularly kept in captivity and come in close contact with humans. In this respect, birds of prey represent a bridging species and may pose a risk of transmitting the virus to humans or to other captive avian species, including poultry.

In the past, HPAI rarely occurred in birds of prey and only in isolated cases. In 2000, Manvell et al. (*5*) isolated influenza virus (H7N3) from a Peregrine falcon (*Falco peregrinus*) kept as a falconry bird in the United Arab Emirates. In the same year, during an HPAI (H7N7) outbreak in poultry in Italy, an avian influenza virus of H7 subtype was isolated from a Saker falcon (*Falco cherrug*) (*6*). Both birds showed depression and died, but other pathogens (e.g., *Pasteurella* sp.) were detected as well.

During recent influenza (H5N1) outbreaks, increasing numbers of birds of prey were reported to be infected. HPAI virus (H5N1) was isolated from Hodgson’s hawk eagles (*Spizaetus nipalensis*) confiscated at an airport (*7*) and from a Saker falcon (*8*) in Saudi Arabia. During the influenza (H5N1) outbreak among wild birds in Germany, 36 (10.5%) birds with positive influenza (H5N1) results were birds of prey, represented by common buzzards (*Buteo* *buteo*), peregrines (*F. peregrinus*), and kestrels (*Falco tinnunculus*), as well as European eagle owls (*Bubo bubo*), which were found dead (*9*). Diseased free-ranging birds of prey infected with influenza (H5N1) were also reported by several other countries. In March 2007, influenza virus (H5N1) was isolated from falcons in Kuwait (www.poultrymed.com).

Although it is obvious that birds of prey can be infected with HPAI viruses, the pathogenic potential in these species remains unclear. Free-ranging birds frequently suffer from other concurrent diseases or starvation, and captive birds undergo stressful periods due to rearing conditions or training. These situations may immunocompromise the birds, leading to increased vulnerability. However, their potential to shed virus after infection, which is important for virus transmission, potentially also to humans, remains unclear. Clinical signs, pathologic and histopathologic alterations, and tissue tropism of the virus after a controlled infection have not been investigated. This knowledge is needed for a better understanding of HPAI in nondomestic birds, especially for subtype H5N1, which poses a higher risk to humans than do other avian influenza viruses (*10*–*12*).

Collections of birds of prey are of high commercial and species conservation value; therefore, protection from HPAI is important. Vaccination might reduce the risk for virus transmission by reducing virus shedding, as has been shown in chickens (*13*,*14*). Ultimately, an interruption of virus transmission between and within avian collections would be invaluable for controlling disease, especially in populations of rare species as exemplified by many bird of prey species (*15*).

Vaccination with commercially available inactivated vaccines based on avian influenza virus subtype H5 can confer clinical protection and reduce virus shedding after infection (*16*). Implementation of DIVA (Differentiating Infected from Vaccinated Animals) strategies have been attempted (*17*). Response to vaccination of zoo birds with an AI H5N2 (*18*) or H5N9 (*19*) subtype inactivated vaccine varied considerably among species with respect to peak titers and persistence of specific antibodies. Some species mounted antibodies after the first round of vaccination; others had detectable titers only after a second dose or never produced detectable antibody levels (pelicans [*Pelicanus* spp.] and owls [*B. bubo, Tyto alba*]) (*20*). The authors demonstrated peak hemagglutination inhibition (HI) titers of 2,048 within 2–4 weeks after booster vaccination in bar-headed geese (*Anser indicus*); most other species yielded titers of only 64 to 512 during the same time. Several species, such as the Egyptian goose (*Alopochen aegyptiacus*) and peafowl (*Acryllium vulturinum*), still had antibody titers of 32 to 128 by 6 months after vaccination, while spur-winged geese (*Plectropterus gambensis*) failed to show titers after that time. Such a variation between species was also observed after vaccination of different waterfowl and wader species (*21*).

No detailed information is available about antibody responses and protection after vaccination against HPAI in falcons. Therefore, we analyzed the susceptibility of falcons to an influenza (H5N1) field virus under controlled conditions and evaluated the efficacy of vaccination of falcons with an inactivated influenza (H5N2) vaccine and its effect on epidemiologically relevant parameters. The trial was approved under government registration numbers G 0072/06 and LVL M-V/TSD/7221.3-1.1-45/05 (with expansion LVL M-V/TSD/7221.3-1.1-37/06).

## Materials and Methods

### Animals

Fifteen juvenile female Gyr-Saker *(F. rusticolus* × *F. cherrug*) hybrid falcons were obtained from 1 breeder. The birds received an intensive health evaluation, which included a general examination, radiographs, laparoscopy, blood cell count, blood chemistry analysis, and parasitologic examination; all results were within normal limits. The falcons were perched according to standard falconry techniques during the vaccination trial (*22*). For challenge infection, the animals were kept individually in stainless steel cages located in negatively pressurized isolation rooms within Biosafety Level 3 facilities. Seven 1-day-old chicks obtained from a disease-free stock, were provided to each bird each day as feed. Unconsumed chicks were removed to measure the daily feed intake of each bird.

### Vaccination

Ten falcons were vaccinated (nos. 1–5 intramuscularly and nos. 6–10 subcutaneously) with 0.5 mL (hemagglutinating titer >16) of influenza (H5N2) inactivated vaccine (Intervet, Unterschleissheim, Germany) based on strain A/duck/Potsdam/1402/86; they were revaccinated with the same dose and by the same route 4 weeks later. As a negative control, 5 nonvaccinated falcons were kept with the vaccinated birds.

Before the first vaccination and in weekly intervals until 8 weeks after initial vaccination, individual blood samples were collected from the metatarsalis plantaris superficialis medialis vein directly into a serum tube (Sarstedt, Nümbrecht, Germany) by using a 0.7-mm × 30-mm needle (Sterican, Luer-Lock, Braun Melsungen, Germany). The serum was separated and tested for H5-specific antibodies by the HI test, with low pathogenic avian influenza subtype H5N2 (A/duck/Potsdam/619/85) as antigen according to standardized methods (*23*). A cloacal swab was also examined for influenza A virus RNA by using real-time reverse transcription–PCR (RT-PCR) targeting an M gene fragment to exclude a concurrent infection (*24*).

### Challenge Infection

Five months after the initial vaccination, 5 falcons randomly selected from the 10 vaccinated birds (nos. 1, 2, 5, 8, 9) and 5 nonvaccinated control birds (nos. 11–15) were challenged with 10^6.0^ 50% egg infectious dose (EID_50_) of influenza strain A/*Cygnus cygnus*/Germany/R65/2006, a highly pathogenic H5N1 strain that was isolated from a dead whooper swan (*Cygnus cygnus*) during an outbreak of HPAI virus (H5N1) among wild birds in Germany (*25*). Each bird received 1 mL cell culture medium by the oculo-oronasal route. The falcons were observed daily for 11 days after challenge. At the end of the trial, surviving birds were humanely killed. A serum sample was obtained by using the above-described method just before challenge and, for surviving birds, on the last day of the trial. The serum was used for the detection of antibodies against H5 by the HI test (see above) by using 2 different antigens (challenge and vaccine strain). Before challenge and at days 1, 2, 4, 7, and 11 after challenge, an oropharyngeal and a cloacal swab were taken for a semiquantitative detection of avian influenza virus–specific RNA by using a real-time RT-PCR targeting an M gene fragment as recommended in the Diagnostic Manual for Avian Influenza issued by the European Commission (*26*) and described by Spackman et al. (*24*). The method has been improved by using an internal control in parallel in a duplex reaction (*27*). In addition, virus isolation in embryonated chicken eggs was attempted as described by Werner et al. (*28*). Isolated viruses were characterized as HPAI virus (H5N1) by subtype-specific real-time RT-PCRs (*26*) and by a pathotype-specific real-time RT-PCR (*29*).

### Gross, Histopathologic, and Immunohistochemical Examinations

Necropsies were performed immediately after death. Samples of nasal cavity, trachea, lung, heart, cerebellum, cerebrum, spinal cord, proventriculus, small and large intestine, liver, pancreas, spleen, skin, and kidney were collected and either snap frozen or formalin fixed (48 h) and processed for paraffin embedding according to standardized procedures. For histopathologic examination, paraffin wax sections (3 µm) were dewaxed and stained with hematoxylin and eosin. Immunohistochemical examination for influenza virus A nucleoprotein (NP) was performed according to Klopfleisch et al. (*30*). Briefly, dewaxed sections were incubated with a rabbit anti-NP serum (1:500). As secondary antibody, biotinylated goat anti-rabbit IgG1 (Vector, Burlingame, CA, USA) was applied. By means of the avidin-biotin-peroxidase complex method, a bright red signal was produced. Positive and negative control tissues of chickens that had been infected experimentally with HPAI virus (H5N1) were included. Tissues from the central nervous system (CNS), small intestine, pancreas, trachea, and lung were used for real-time RT-PCR and for virus isolation.

## Results

### Immune Response

During the entire trial, control birds remained negative for avian influenza virus H5-specific antibodies. In addition, influenza A virus RNA was not detected in any of the cloacal swabs. No adverse clinical effects were detected as a result of application of the 2 vaccine doses.

**Figure 1:**
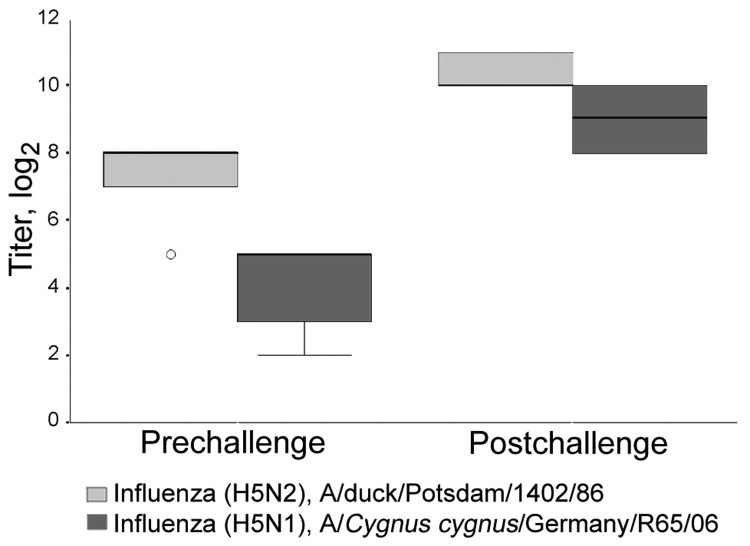
Titers (log_2_) of hemagglutination-inhibiting antibodies of 5 vaccinated Gyr-Saker hybrid falcons before and 11 days after challenge with 10^6.0^ 50% egg infectious doses of the highly pathogenic avian influenza strain A/*Cygnus cygnus*/Germany/R65/06 (H5N1), tested against antigen of the challenge virus and the low pathogenicity avian influenza vaccine strain A/duck/Potsdam/1402/86 (H5N2). Open circle, individual outlier.

Nine of the 10 vaccinated birds mounted homologous H5-specific antibodies 3 weeks after the first vaccination; titers increased significantly after the booster vaccination (Table 1). The remaining bird (no. 5) showed a detectable titer of 8 only 6 weeks after initial vaccination (2 weeks after booster vaccination); HI titer for this bird remained at 8. Differences in titer development according to route of vaccination were not detected (Table 1). Clinical signs (i.e., decreased food intake or worsening general condition) were not observed in any of the vaccinated birds. HI titers against the heterologous challenge strain A/*Cygnus cygnus*/Germany/R65/2006 at the time of challenge are shown in Figure 1. The nonvaccinated birds remained seronegative.

**Table 1:** Titers (log_2_) of hemagglutination-inhibiting antibodies against homologous influenza H5 antigen in 10 Gyr-Saker hybrid falcons after vaccination*

Falcon no., vaccination route	Titer at 0–8 weeks after vaccination								
	0†	1	2	3	4‡	5	6	7	8
1, IM	0	0	0	2	4	5	6	6	7
2, IM	0	0	0	2	3	4	6	8	9
3, IM	0	0	0	2	5	6	7	8	8
4, IM	0	0	0	3	4	4	6	7	6
5, IM	0	0	0	0	0	0	3	3	3
6, SC	0	0	0	3	4	5	6	8	7
7, SC	0	0	0	2	3	4	6	8	7
8, SC	0	0	0	3	3	4	5	6	7
9, SC	0	0	0	2	4	6	6	7	7
10, SC	0	0	0	3	4	5	6	6	6

*Vaccination with an inactivated influenza (H5N2) vaccine (strain A/duck/Potsdam/1402/86) at a dose of 0.5 mL containing >4 log_2_ hemagglutinating units. IM, intramuscular; SC, subcutaneous. †Time of first vaccination. ‡Time of booster vaccination.

### Gross, Histopathologic, and Immunohistochemical Response to Challenge

All nonvaccinated birds died after challenge with HPAI virus (H5N1). The first falcon died on day 3 postchallenge, 3 died on day 4, and the rest died at day 5. Of these, 4 had reduced food intake starting from the day of challenge, and 3 had a slightly bloody tracheal exudate detectable the day after exposure. One bird died with no clinical signs.

All vaccinated birds survived. For 2, food intake was slightly reduced 1 day after challenge. No other vaccinated bird exhibited clinical signs. By 11 days after challenge, the titers of the vaccinated birds increased to 2,048 against the antigen used for vaccination and 1,024 against the challenge strain (Figure 1).

**Figure 2:**
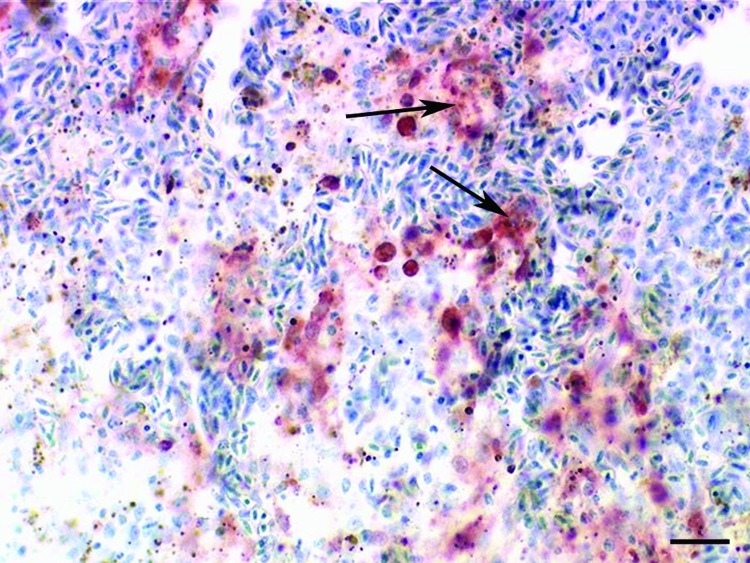
Immunohistochemical demonstration of influenza A virus antigen (red, see arrows) in numerous splenic macrophages of a falcon after challenge with 10^6.0^ 50% egg infectious doses of the highly pathogenic avian influenza strain A/*Cygnus cygnus*/Germany/R65/06 (H5N1). Avidin-biotin-peroxidase complex method. Bar = 25 μm.

Necropsy showed multifocal acute hemorrhagic necrosis in the pancreas of 3 birds that died spontaneously and moderate to severe splenic hyperplasia in 3 birds. Histopathologic examination of the cerebellum, cerebrum, spinal cord, pancreas, spleen, and kidney of the nonvaccinated birds showed multifocal acute cellular degeneration and necrosis associated with minimal to mild infiltration of few heterophils and detection of HPAI virus antigen (Figure 2). Furthermore, antigen was present in the nasal cavity, trachea, bronchial epithelium, and gastrointestinal tract but not in the liver and skin. None of the euthanized vaccinated birds exhibited any gross or histologic lesions or presence of antigen in any of the tissues.

### Virus Excretion after Challenge

**Figure 3:**
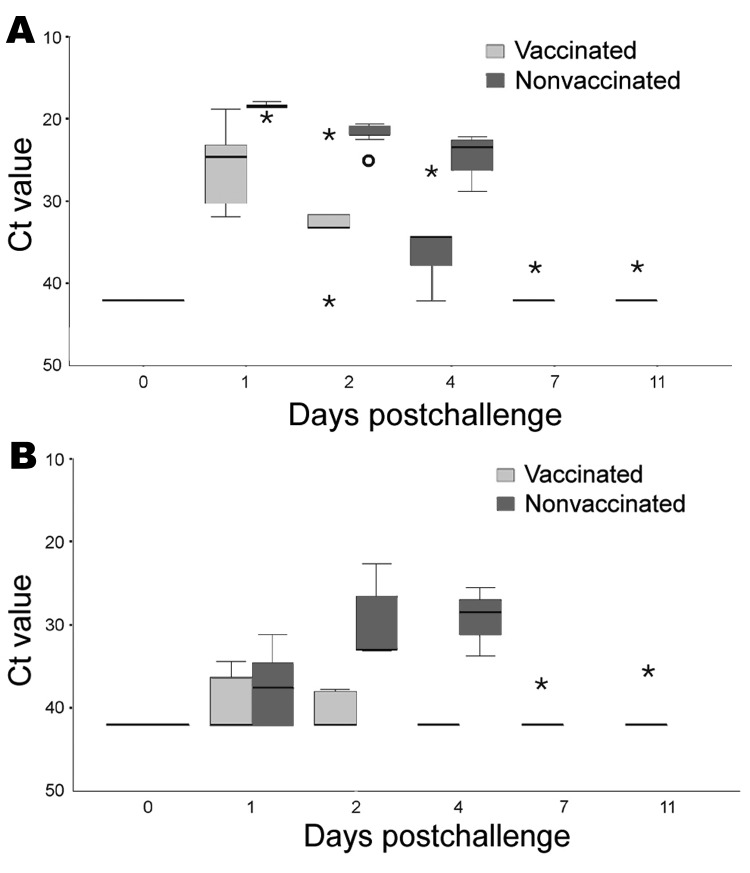
Detection of viral RNA by real-time reverse transcription–PCR (RT-PCR) from oropharyngeal (A) and cloacal (B) swabs of 5 vaccinated and 5 nonvaccinated falcons after challenge with 10^6.0^ 50% egg infectious doses of the highly pathogenic avian influenza virus strain A/*Cygnus cygnus*/Germany/R65/06 (H5N1). Y axis shows cycle-of-threshold (Ct) values of real-time RT-PCRs detecting an M gene fragment in individual swab samples of each animal. Asterisks represent extreme values; open circles show individual outliers; black bars within boxes indicate medians.

In the nonvaccinated falcons, after challenge infection viral RNA was detectable in all oropharyngeal swabs. Virus was also isolated from the pooled oropharyngeal swabs of these birds taken on the same days (Figure 3; Table 2). At day 1 postchallenge, viral RNA was detected in the cloacal swabs of 3 birds, although virus isolation failed. From day 2 postchallenge, all falcons demonstrated the presence of viral RNA and infectious virus in cloacal swabs (Figure 3; Table 2).

**Table 2:** Excretion of infectious highly pathogenic avian influenza virus (H5N1) in vaccinated and control falcons after challenge

Vaccination status	Excretion route*	Days postchallenge†					
		0	1	2	4	7	11
Vaccinated	Oropharyngeal	<0.5	4.4	1.2	<0.5	<0.5	<0.5
Nonvaccinated	Oropharyngeal	<0.5	5.4	4.4	2.0	No data	No data
Vaccinated	Cloacal	<0.5	<0.5	<0.5	<0.5	<0.5	<0.5
Nonvaccinated	Cloacal	<0.5	<0.5	3.0	2.7	No data	No data

*Pooled samples of all birds in the group were examined. †Data represent log_10_ of 50% egg infectious dose per mL.

In the vaccinated falcons, 1 day after challenge viral RNA was detectable in all oropharyngeal swabs. At day 2 postchallenge, 1 falcon became negative for viral RNA, and at days 7 and 11, only 1 bird remained positive for viral RNA (Figure 3; Table 2). Virus isolation from a pool of all oropharyngeal swabs of all 5 vaccinated birds taken from day 1 postchallenge demonstrated a virus titer of 4.4 log_10_ EID_50_/mL at day 1 postchallenge and 1.2 log_10_ EID_50_/mL at day 2 postchallenge. From day 4 on, virus could no longer be isolated from the pooled oropharyngeal swabs. Viral RNA was only occasionally detected in cloacal swabs and completely absent in 1 bird (Figure 3, Table 2). Virus could not be isolated from any of the pools of the cloacal swabs.

### Virus in Tissues

In the nonvaccinated falcons, high loads of viral RNA were detected in the CNS, duodenum, pancreas, trachea, and lung of all control birds that died after challenge (Table 3). Virus isolation from these samples was not attempted.

**Table 3:** Viral RNA in tissues of 5 vaccinated and 5 nonvaccinated falcons*

Falcon no.,vaccination route	RNA in tissue, ct value†				
	CNS	Duodenum	Pancreas	Trachea	Lung
Vaccinated					
1, IM	36.12‡	>40.00	38.84‡	>40.00	>40.00
2, IM	38.65‡	>40.00	>40.00	>40.00	>40.00
5, IM	34.22‡	36.14‡	>40.00	33.44‡§	36.94‡
8, SC	>40.00	IH	>40.00	38.71‡	32.85‡§
9, SC	>40.00	IH	IH	29.01§	IH
Nonvaccinated					
11	18.22	25.84	19.70	19.09	20.75
12	23.79	23.37	14.67	19.07	18.95
13	12.85	26.22	20.13	19.82	16.95
14	11.52	21.66	17.14	16.70	15.55
15	14.13	16.31	19.91	14.61	18.04

*Viral RNA detected by real-time reverse transcription–PCR (RT-PCR) in vaccinated falcons euthanized 11 days postchallenge and in nonvaccinated falcons that died after challenge infection with 10^6.0^ 50% egg infectious doses of highly pathogenic avian influenza virus strain A/*Cygnus cygnus*/Germany/R65/06 (H5N1). Ct, cycle of threshold; CNS, central nervous system; IM, intramuscular; SC, subcutaneous; IH, inhibited (samples extracted twice with the QIAGEN [Hilden, Germany] Viral RNA kit or Trizol [Invitrogen, Carlsbad, CA, USA]). †Real-time RT-PCR results are presented as ct values. Ct values >40 are scored as negative. ‡Virus isolation attempted in embryonated chicken eggs. §Virus was isolated from individual tissue sample.

In the vaccinated falcons, low to moderate loads of viral RNA were demonstrated in the brain and trachea of 3 birds that were euthanized on day 11 postchallenge, in the lung of 2 birds, in the duodenum of 1 bird, and in the pancreas of 1 bird. However, virus was isolated from the trachea of only 2 birds and from the lung of 1 (Table 3). The viral RNA load was as much as 6 log_10_ lower than that of nonvaccinated animals.

## Discussion

Our study is the first, to our knowledge, to demonstrate that falcons are highly susceptible to HPAI virus (H5N1) as exemplified by strain A/*Cygnus cygnus*/Germany/R65/2006; all nonvaccinated birds died within 5 days after challenge. Clinical signs were mild and indicated only by a reduced food intake, which is not considered very obvious because falcons typically do not eat every day. These signs will not be seen in free-ranging birds and may be overlooked in captive animals. However, under natural conditions, more pronounced clinical signs may develop because stress situations and concurrent diseases are more likely than in captivity. Considering virus replication in the CNS, as demonstrated by immunohistochemical examination, CNS disturbances such as ataxia and disorientation might have ensued, although this is difficult to verify when birds are not allowed to fly.

The slightly bloody exudate from the trachea, noted for 3 birds at day 1 postchallenge, may pass unnoticed under field conditions. On the basis of the inconspicuous clinical signs, precisely defining the length of the incubation period is difficult. Gross lesions noted at necropsy were only mild and restricted to the pancreas and, thus, may be overlooked during routine necropsy when influenza is not suspected. The striking alterations of the pancreas are important as they were found macroscopically in 3 of the 5 birds and histopathologically in all 5 birds that died. Such lesions have also been described in mute (*C. olor*) and whooper swans (*C. cygnus*) (*31*), in passerines and budgerigars (*32*), and in emus and geese (*33*). The systemic virus distribution parallels that noted in water fowl during the 2006 outbreak on the Baltic Sea coast (*31*). Nevertheless, carnivorous birds, including buzzards, affected during an outbreak in Germany in 2006 displayed mainly a severe infection of the CNS without systemic virus distribution (unpub. data). The lack of antigen detection in the vaccinated falcons at day 11 postchallenge parallels the minimal virus shedding of the vaccinated falcons. Nevertheless, infection of cells at the site of inoculation can only be excluded by immunohistochemical examination of vaccinated animals during the first days after challenge.

All nonvaccinated falcons shed virus from the oropharynx and cloaca until death. Oropharyngeal shedding peaked at day 1 postchallenge, which might be related to reisolation of inoculum, and decreased toward day 4 postchallenge. The peak of cloacal excretion was at day 2 postchallenge, as reported for chickens (*14*). These findings demonstrate that after infection with influenza A (H5N1) of Asian origin, oropharyngeal swabs may be superior to cloacal swabs for diagnosing infection under field conditions. Duration of virus excretion before death was very short. Therefore, falcons may not play a major role in spreading the pathogen within or between countries, although this possibility cannot be excluded. Moreover, infected birds, like these falcons, may not be able to migrate long distances. However, because they shed a considerable amount of virus for a short time concomitant with virtual absence of overt clinical signs, captive infected falcons may pose a substantial risk for humans and other birds of high commercial and species conservation value. Therefore, measures to reduce this risk are of great importance, especially because depopulation of such birds is not a well-accepted option.

Vaccination of poultry, at least in experimental settings, can reduce virus shedding significantly after challenge, depending on the amount of antigen in the vaccine and the antigenic relationship between vaccine and virulent field virus (*13*,*14*,*34*,*35*). This study shows that vaccination is also an option in falcons. It is safe; no adverse clinical reactions were observed. High titers of specific HI antibodies were induced in most vaccinated animals and persisted for at least 5 months, which indicates that biannual revaccination may suffice. However, as in chickens, sterile immunity could not be induced as shown by continuous detection of virus excretion, particularly from the oropharynx, in vaccinated falcons after challenge infection. However, virus excretion was drastically reduced in vaccinated birds compared with nonvaccinated birds and could be detected only by sensitive real-time RT-PCR. With respect to the marked differences of virus excretion between vaccinated and nonvaccinated falcons, we note that a difference of approximately 3.3 cycle-of-threshold values corresponds to 1 log_10_ of viral nucleic acid copies (*36*). Figure 3A shows that in oropharyngeal swabs from nonvaccinated falcons, up to 3–4 log_10_ more viral RNA copies are present than in swabs from vaccinated falcons. The failure to isolate challenge virus from excretions of vaccinated falcons raises the question of the epidemiologic importance of the presence of viral RNA in oropharyngeal swabs (*13*,*14*). Therefore, vaccination is considered to be an important tool to prevent further major outbreaks (*34*). Additionally, the bird-to-human infection route of HPAI seems to require a high amount of excreted virus as well as close contact (*37*), which seems much more difficult to achieve with vaccinated birds. Although residual infectious virus persisted in organs of a few vaccinated birds until day 11 postchallenge, whether viral loads are sufficient for efficient transmission remains unclear. Because no viral RNA could be detected in the oropharyngeal swabs of 2 of these birds, this, however, appears to be unlikely.

In conclusion, we have demonstrated that falcons are highly susceptible to HPAI (H5N1) but can be protected from clinical disease and death by vaccination with a heterologous inactivated vaccine administered intramuscularly or subcutaneously. Virus shedding was grossly reduced after vaccination, thereby decreasing risk for further virus transmission to other avian species as well as to humans. However, use of vaccine will require the establishment of an appropriate surveillance program that includes use of serologic testing, PCR, and sentinel birds.
